# Transcutaneous electrical acupoint stimulation combined with electroacupuncture promotes rapid recovery after abdominal surgery: Study protocol for a randomized controlled trial

**DOI:** 10.3389/fpubh.2022.1017375

**Published:** 2022-11-14

**Authors:** Hao Li, Chen Du, Lingyun Lu, Xiangyun Hu, Huiming Xu, Ning Li, Hong Liu, Qian Wen

**Affiliations:** ^1^Center for Integrative Medicine, Sichuan University West China Hospital, Chengdu, China; ^2^Party Committee Office, Sichuan University West China Hospital, Chengdu, China

**Keywords:** transcutaneous electrical acupoint stimulation, electroacupuncture, rapid recovery, abdominal surgery, randomized controlled trial

## Abstract

**Introduction:**

The most frequent complications after abdominal surgery include a decrease or loss of appetite, abdominal distension, abdominal pain caused by reduced gastrointestinal motility, anal arrest with intestinal distension and defecation, and nausea and vomiting due to anesthetic and opioid analgesic administration. These complications severely affect postoperative recovery, prolong hospital stay, and increase the financial burden. The objective of this study is to investigate the efficacy and safety of three acupoint stimulation modalities (electroacupuncture [EA], transcutaneous electrical acupoint stimulation [TEAS], and transcutaneous acupoint electrical stimulation combined with EA [TEAS+EA]), and two EA instrument waveforms (continuous wave and dilatational wave) for rapid recovery after abdominal surgery.

**Methods and analysis:**

A total of 560 patients will be recruited and randomly allocated to receive one of the following seven interventions: continuous wave EA, continuous wave TEAS, continuous wave TEAS + EA, dilatational wave EA, dilatational wave TEAS, dilatational wave TEAS + EA, and a control. For this study, continuous waves at 2 Hz, and dilatational waves at 2/50 Hz would be selected. The points to be stimulated by EA are the bilateral Neiguan (PC6), Hegu (LI6), Zusanli (ST36), Shangjuxu (ST37), and Xiajuxu (ST39), and TEAS would stimulate the bilateral Liangmen (ST21) and Daheng (SP15). The control group will neither receive EA nor TEAS. All patients will undergo an enhanced recovery plan after surgery and be provided with standardized perioperative management. Treatment will start on the first postoperative day and be administered once daily in the morning until the patient regains spontaneous bowel movements and can tolerate oral intake of solid food. The primary outcome is a composite of time to first defecation and time to tolerance of a solid diet. Secondary outcomes include time to first exhaustion; time of first defecation; time of tolerance of a solid diet; time to the first ambulation; length of hospital stay from surgery to discharge; visual analog scale score for postoperative daily pain, nausea, and vomiting; incidence of postoperative complications; and treatment acceptability.

**Discussion:**

This study will compare the efficacy and safety of three acupoint stimulation methods and two EA instrument waveforms for rapid recovery after abdominal surgery.

**Trial Registration:**

Chinese Clinical Trial Registry (http://www.chictr.org.cn), ChiCTR2100043883.

## Introduction

Postoperative complications of abdominal surgery include gastrointestinal dysfunction, pain, nausea, and vomiting (1, 2). These complications adversely affect the patient's quality of life, prolong the length of hospital stay, increase hospitalization costs, and the risk of needing a second operation (3). Therefore, reducing postoperative complications and promoting rapid recovery after surgery have been widely studied (4).

An Increasing amount of clinical evidence supports the use of acupuncture for enhanced recovery after surgery (ERAS) (5–7). Nevertheless, there is no unified standard for the use of specific acupuncture methods, acupoint selection, and electroacupuncture (EA) instrument parameters (8, 9). The majority of previous studies evaluating the use of acupuncture during postoperative rehabilitation after abdominal surgery have utilized distal limb acupoints (10, 11). This may be related to the surgical wound after abdominal surgery and changes in the structure and state of the abdominal organs which affect the acupuncture procedure and its safety. However, emerging evidence in recent years has indicated that acupuncture applied to select abdominal or limb acupoints can lessen the degree of abdominal pain and distension (12, 13). The use of abdominal meridian points may be particularly effective in decreasing abdominal pain (14, 15). This study is based on many early clinical practices and on the application of EA at meridian points in the distal extremities where efficacy was compared with the addition of a safer transcutaneous electrical acupoint stimulation (TEAS) of the abdominal acupoints.

Stimulation parameters are critical factors for the effectiveness of EA, as different stimulation parameters will bring about different therapeutic effects (16, 17). The use of a continuous wave in EA results in muscle contraction, exciting sensation, and motor nerve. The continuous wave is the most commonly used waveform to strengthen the effect of acupuncture in the clinic. In contrast, dilatational waves can increase metabolism, promote blood circulation, and improve muscle weakness and other functions. Dilatational waves are frequently used to facilitate postoperative rehabilitation (18). To date, no studies have compared the effectiveness of these two waveforms in promoting rapid rehabilitation after abdominal surgery (19).

Therefore, the primary purpose of this randomized controlled study is to compare the efficacy and safety of three acupoint stimulation methods and two EA instrument waveforms in facilitating rapid recovery after abdominal surgery, with the overarching aim of informing the development of an objective and unified standard for acupuncture use to facilitate rapid postoperative recovery.

## Methods and analysis

### Study design

This is a prospective, single-center, parallel, single-blind, randomized controlled trial. Effectiveness and safety will be compared among three acupoint stimulation methods (EA, TEAS, TEAS+EA) and two EA instrument waveforms (continuous waves, dilatational waves) to determine their suitability for promoting them in postoperative rehabilitation. This study will be conducted in accordance with the principles of the Declaration of Helsinki. The “Standard Protocol Items: Recommendations for Interventional Trials” checklist is provided in Supplementary Material 1.

### Recruitment

**Figure 1:**
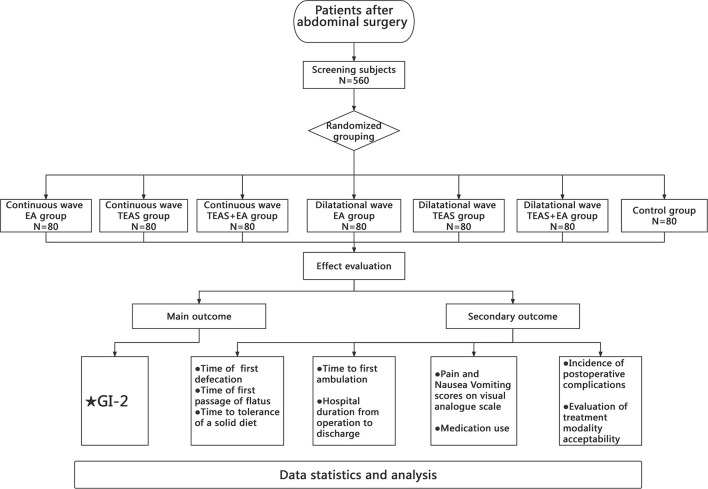
Flowchart of the study protocol.

This trial will be conducted from 1 April 2021 to 31 March 2023, at West China Hospital of Sichuan University. A total of 560 patients who have undergone abdominal surgery (i.e., hepatobiliary, gastrointestinal, renal, or bladder tumor resection) and meet the study inclusion and exclusion criteria will be enrolled in this study. All patients will be requested to provide written informed consent prior to study participation. Figure 1 presents the study flow chart.

### Inclusion criteria

Patients will be included if they (1) are aged 18–70 years old, male or female; (2) have undergone resection of hepatobiliary, gastrointestinal, renal, or bladder tumors under general anesthesia; and (3) participate voluntarily and provide written informed consent.

### Exclusion criteria

The exclusion criteria would comprise the following: (1) the surgical incision was made through the abdominal acupoints selected for this study; (2) local skin infection is evident at the selected acupoints; (3) patients are unable to understand or cooperate with the assessments (e.g., visual analog scale [VAS]); (4) metal allergy or severe fear of acupuncture, TEAS, or EA; (5) uncontrolled diabetes, severe coagulopathy, or cardiac, central nervous system, or psychiatric disorders; (6) pacemaker; and (7) concurrent enrolment in other research trials.

### Withdrawal criteria

Patients will be withdrawn from the study if they (1) experience serious adverse events, (2) have a serious complication or illness during the study that requires urgent action; or (3) withdraw informed consent.

### Randomization and blinding

This study has a single-blind design. Patients will be unaware of their group allocation, which will only be known to the lead investigator and acupuncture physician. The randomization sequence and allocation ratio (1:1:1:1:1:1) will be generated using the statistical software SPSS 26.0. Group allocation will be concealed using opaque envelopes, which will be distributed sequentially after patient enrolment.

### Intervention

**Figure 2:**
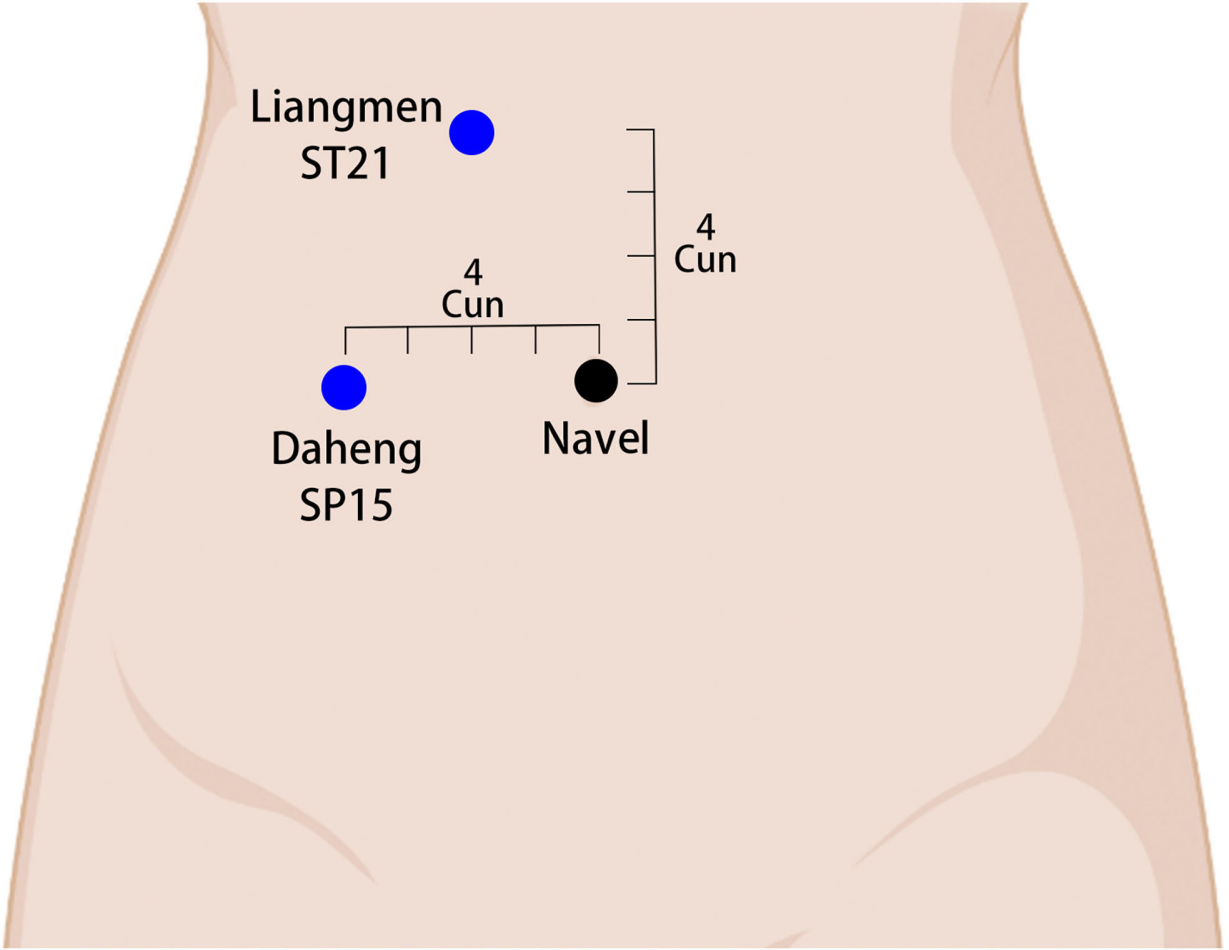
Localization of abdomen acupoints and electrode connection. ST21 (Liangmen) in the upper abdomen, 4 cun above the navel and 2 cun from the anterior midline. SP15 (Daheng) in the ventral part, 4 cun from the navel. A set of electrodes is attached at the blue dot (transcutaneous electrical acupoint stimulation).

**Figure 3:**
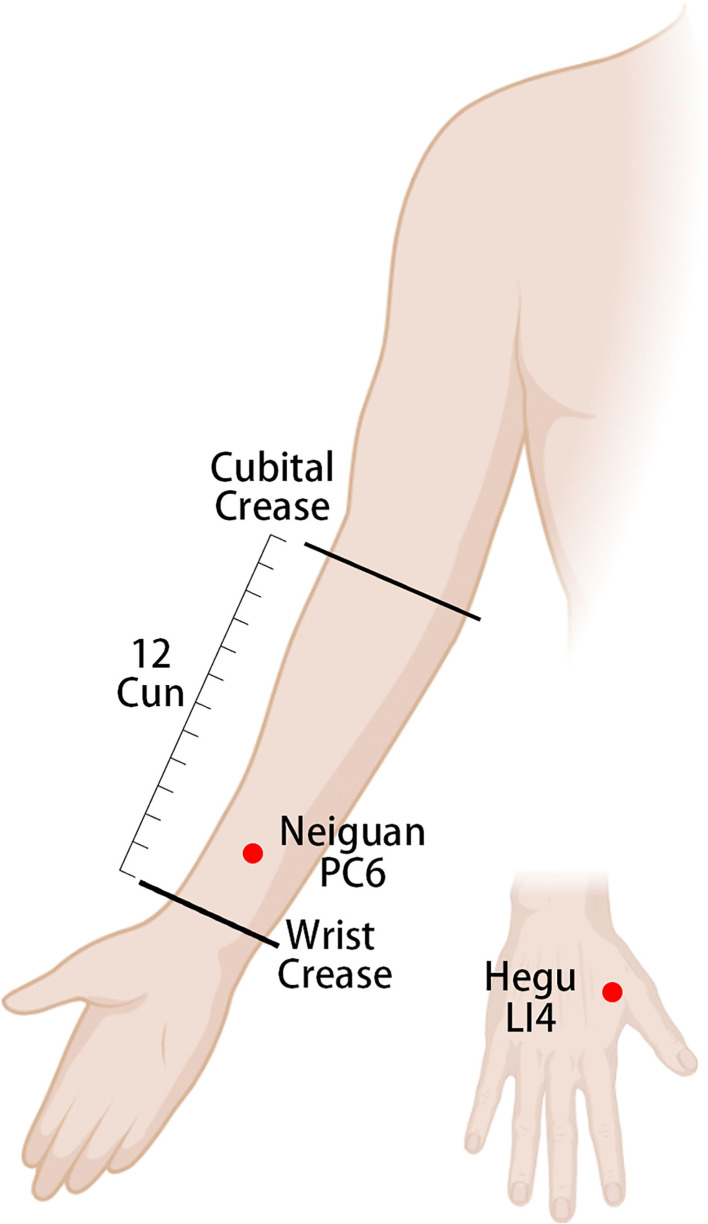
Location of upper extremity acupoints and electrode connection. PC6 (Neiguan) on the volar aspect of the forearm, 2 cun on the wrist crease, and between the palmaris longus tendon and the flexor carpi radialis tendon. LI4 (Hegu) between the first and second metacarpals, at the midpoint of the radial aspect of the second metacarpal. A set of electrodes is attached at the red dot (electroacupuncture).

**Figure 4:**
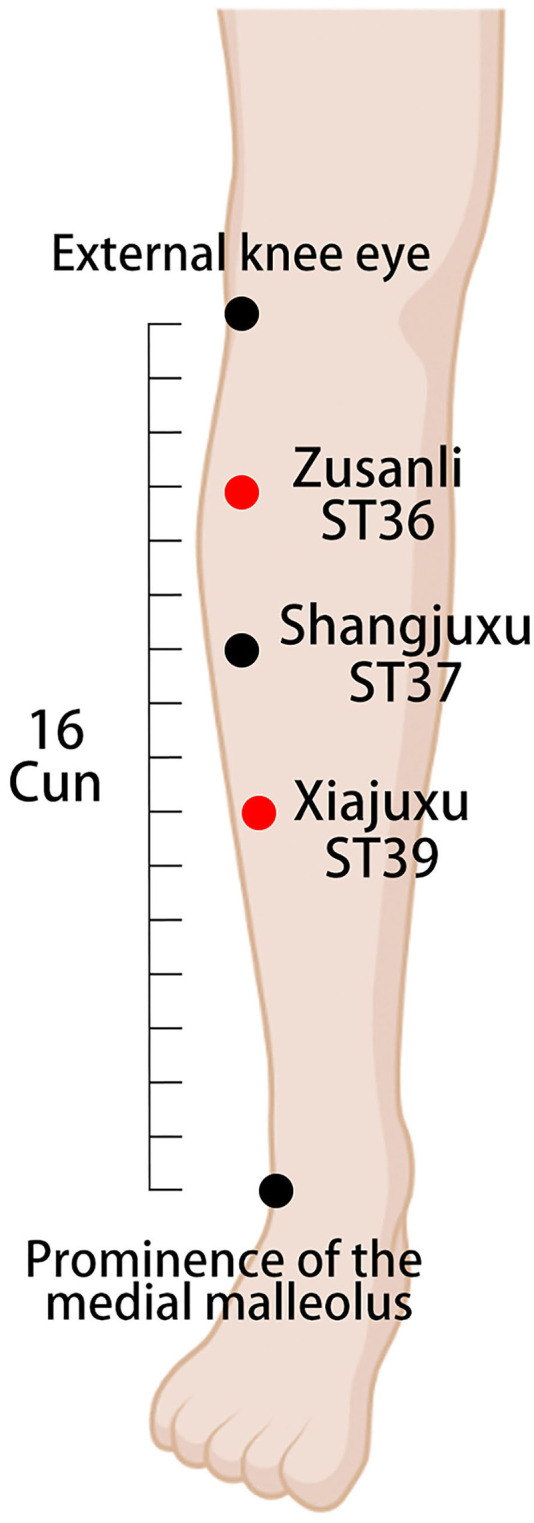
Location of lower extremity acupoints and electrode connection. ST36 (Zusanli) on the lateral lower leg, 3 cun under the external knee eye. ST37 (Shangjuxu) on the lateral lower leg, 6 cun under the external knee eye and 3 cun under the ST36 (Zusanli) acupoint. ST39 (Xiajuxu) on the lateral lower leg, 9 cun under the external knee eye. A set of electrodes is attached at the red dot (electroacupuncture).

Each group will receive an Enhanced Recovery After Surgery (ERAS)-standardized perioperative management (20). The acupoints used in the treatment group are referenced from the China National Standard Nomenclature and Location of Meridian Points (GB 12346-2021) (21). The selected acupoints and their locations are shown in Figures 2–4. The Hwato SDZ-V (Suzhou Medical Supplies Factory) EA apparatus will be used, and the current intensity will be adjusted to the tolerance level of the patient. Each treatment will last for 30 min and the initial session will commence on the first postoperative day. Treatment sessions will be provided once daily in the morning until the patient regains spontaneous flatus and can tolerate oral intake of solid food. All acupuncture maneuvers will be performed independently by the same acupuncturist, who has at least 5 years of work experience. The acupuncturist will not be replaced at any point during the study.

#### Continuous wave EA (cEA) group

EA will be used to stimulate the bilateral Neiguan (PC6), Hegu (LI6), Zusanli (ST36), Shangjuxu (ST37), and Xiajuxu (ST39). The current frequency will be a continuous wave at 2 Hz.

#### Continuous wave TEAS (cTEAS) group

TEAS will be used to stimulate the bilateral Liangmen (ST21) and Daheng (SP15). The current frequency will be a continuous wave at 2 Hz.

#### cTEAS + EA group

EA will be used to stimulate the bilateral Neiguan (PC6), Hegu (LI6), Zusanli (ST36), Shangjuxu (ST37), and Xiajuxu (ST39). TEAS will be used to stimulate the bilateral Liangmen (ST21) and Daheng (SP15). The current frequency will be a continuous wave at 2 Hz.

#### Dilatational wave EA (dEA) group

EA will be used to stimulate the bilateral Neiguan (PC6), Hegu (LI6), Zusanli (ST36), Shangjuxu (ST37), and Xiajuxu (ST39). The current frequency will be a dilatational wave at 2/50 Hz.

#### Dilatational wave TEAS (dTEAS) group

TEAS will be used to stimulate the bilateral Liangmen (ST21) and Daheng (SP15). The current frequency will be a dilatational wave at 2/50 Hz.

#### dTEAS + EA group

EA will be used to stimulate the bilateral Neiguan (PC6), Hegu (LI6), Zusanli (ST36), Shangjuxu (ST37), and Xiajuxu (ST39). TEAS will be used to stimulate the bilateral Liangmen (ST21) and Daheng (SP15). The current frequency will be a dilatational wave at 2/50 Hz.

#### Control group

Only ERAS-standardized perioperative management, without TEAS or EA, will be performed in the control group.

All acupoints will be routinely disinfected. The acupoints in the distal limb will be punctured straight through the skin to a depth of 25–30 mm using disposable stainless steel needles (0.25 × 40 mm, Suzhou Jiajian, Jiangsu, China). The needle will then be twisted slightly to achieve the de qi sensation, and the EA apparatus will be connected with a set of electrodes in the ipsilateral Neiguan (PC6), Hegu (LI4), Zusanli (ST36), and Xiajuxu (ST39). The abdominal acupoints will be stimulated with a self-adhesive electrode sheet with electrical conductivity, and the ipsilateral Liangmen (ST21) will be connected with a set of electrodes at Daheng (SP15).

### Outcome measures

#### Primary outcome

The primary outcome is Gastrointestinal-2, which is a composite outcome of time to first defecation and time to tolerance of a solid diet (22).

#### Secondary outcomes

Secondary outcomes comprise the following: (1) time of first spontaneous exhaustion after operation; (2) time of first spontaneous defecation after operation; (3) time of first tolerance to oral intake of solid food after operation; (4) time to first ambulation after surgery; (5) VAS scores for postoperative daily pain and nausea and vomiting; (6) postoperative daily incidence of nausea and vomiting; (7) length of hospital stay, from surgery to discharge; (8) incidence of postoperative complications; and (9) acceptability of acupuncture therapy on a 5-point Likert scale (very acceptable, moderately acceptable, somewhat acceptable, moderately unacceptable, and totally unacceptable).

### Safety evaluation

EA-related safety evaluation during treatment includes the documentation of broken needles, fainting due to needles, intolerable pinprick pain, local hematoma, infection, abscess, and other incidences of discomfort after pinprick. Adverse events will be recorded by the acupuncture physician in a standardized form.

### Sample size

The determination of the sample size was based on the following pre-experimental results for the primary outcome measure, Gastrointestinal-2: cEA, 84.8 ± 36.1 h; cTEAS, 86.8 ± 40.1 h; cTEAS+EA, 70.3 ± 39.3 h; dEA, 78.3 ± 42.2 h; dTEAS, 81.2 ± 44.2 h; dTEAS+EA, 64.1 ± 36.3 h; and control, 110.8 ± 42.3 h. PASS 15 software was used to determine the sample size of 560 patients (80 patients in each group; α = 0.05 [two-sided], β = 0.1 [90% power], with an assumed 20% dropout rate).

### Statistical analysis

The data will be analyzed using SPSS 26.0. All statistical tests will be two-sided, and a *p*-value of less than 0.05 will be considered statistically significant. Measurement data conforming to a normal distribution will be expressed as mean ± standard deviation (x ± s). Comparisons among groups will be performed *via* one-way analysis of variance, using the Student–Newman–Keuls q-test for pairwise comparisons. Variables that do not follow a normal distribution will be analyzed with the Kruskal–Wallis H-test. Categorical and count data will be described as frequency or percentage and compared among groups using the chi-square or Fisher's test.

## Discussion

Although perioperative ERAS measures can facilitate accelerated recovery in patients, there is still much room for improvement in preventing and treating postoperative gastrointestinal dysfunction and analgesia, as well as reducing the length of hospital stay (23–25). Acupuncture can contribute to postoperative multimodal analgesia. Postoperative analgesia is one of the core components of ERAS. While opioids remain the conventional option for postoperative pharmacological analgesia, they are also associated with nausea, vomiting, and other complications. Thus, reducing opioid use can help patients recover sooner. Many studies have been conducted on the mechanism of acupuncture analgesia through electrophysiology, neurochemistry, molecular biology, and brain imaging investigations (26–28). Patient recovery is also adversely affected by postoperative nausea and vomiting (29), which may be effectively prevented by Neiguan (PC6) stimulation (30, 31).

Previous studies have demonstrated the efficacy of acupuncture in rapid postoperative rehabilitation (32). However, there may be concerns that the presence of surgical wounds after abdominal surgery and the possible changes in the structure and state of abdominal organs after abdominal surgery can affect the manipulation and safety of acupuncture. Therefore, previous clinical experience and research for acupuncture treatment after abdominal surgery have primarily selected acupoint stimulation on the distal limbs (24). Studies have shown that Zusanli (ST36), Shangjuxu (ST37), and Xiajuxu (ST39) stimulation can effectively improve gastrointestinal transit by reducing local inflammation of the intestinal musculature (33). Thus, based on the plethora of available clinical evidence for the use of EA at distal extremity acupoints, we have proposed in the present study the adjunctive use of TEAS, which is safer than EA for the stimulation of abdominal acupoints. Moreover, the selected bilateral Liangmen (ST21) and Daheng (SP15) of the abdomen are unconventional locations for incisions during abdominal surgery. Daheng (SP15) is a pair of acupoints belonging to the spleen meridian, and Liangmen (ST21) is a pair of acupoints belonging to the stomach meridian. These acupoints are more convenient to use; they are also antiemetic and promote gastrointestinal peristalsis, relieve abdominal pain, and have other effects (34, 35).

Therefore, this parallel-group randomized controlled trial aims to compare the efficacy and safety of three acupoint stimulation methods and two EA instrument waveforms for facilitating rapid recovery after abdominal surgery. The results of this study will inform the development of an objective and unified standard for EA and TEAS as indispensable components of ERAS.

### Limitation

This trial will not include a sham control arm, and a placebo response and effect analysis is lacking. Nevertheless, some studies on the use of acupuncture for gastrointestinal symptoms have shown that EA may have more significant benefits than sham acupuncture, despite a placebo effect (36, 37).

## Data availability statement

The datasets are not readily available as they are currently under the protection of WCHSU. Data for use or analysis following study completion will be available from the corresponding author on reasonable request.

## Ethics statement

The studies involving human participants were reviewed and approved by the Institutional Review Board of West China Hospital of Sichuan University. The participants provided written informed consent to participate in this study.

## Author contributions

HaL and CD contributed equally to this article. HaL and QW conceived the idea for this study. CD participated in the design and drafted the manuscript. L-yL and H-mX are responsible for recruiting subjects. X-yH and NL contributed to the final version of the manuscript. HoL and QW are responsible for monitoring this study. All authors contributed to the manuscript revision, read, and approved the submitted version.

## Funding

This study is supported by the Sichuan Science and Technology Program (Grant No.: 2021YFS0254).

## Conflict of interest

The authors declare that the research was conducted in the absence of any commercial or financial relationships that could be construed as a potential conflict of interest.

## Publisher's note

All claims expressed in this article are solely those of the authors and do not necessarily represent those of their affiliated organizations, or those of the publisher, the editors and the reviewers. Any product that may be evaluated in this article, or claim that may be made by its manufacturer, is not guaranteed or endorsed by the publisher.

## Abbreviations

cEA: continuous wave electroacupuncture
cTEAS: continuous wave transcutaneous electrical acupoint stimulation
dEA: dilatational wave electroacupuncture
dTEAS: dilatational wave transcutaneous electrical acupoint stimulation
EA: electroacupuncture
ERAS: enhanced recovery after surgery
TEAS: transcutaneous electrical acupoint stimulation
VAS: visual analog scale.
